# Evolution of brain-computer interface technologies for stroke rehabilitation: a bibliometric integration of neural decoding and functional recovery (2016–2025)

**DOI:** 10.3389/fnins.2026.1871816

**Published:** 2026-07-10

**Authors:** Yutong Pan, Yiyang Huang, Mingming Bao, Xiandong Sun

**Affiliations:** 1 Occupational Therapy, Massachusetts College of Pharmacy and Health Sciences, Worcester, MA, United States; 2 Rehabilitation Therapy, Clinical Medical College of Tianjin Medical University, Tianjin, China; 3 Department of Rehabilitation Medicine, Chifeng Municipal Hospital, Chifeng, Inner Mongolia, China; 4 Department of Hypertension Center, Chifeng Municipal Hospital, Chifeng, Inner Mongolia, China

**Keywords:** artificial intelligence, bibliometrics, brain-computer interface, neuroplasticity, rehabilitation, robots, stroke, upper limb

## Abstract

**Introduction:**

Brain-computer interface (BCI) technology represents a critical frontier in neurorehabilitation. This study aims to systematically analyze the global research landscape, hotspot distribution, and evolving trends of BCI interventions for upper limb rehabilitation in stroke survivors between 2016 and 2025.

**Methods:**

Bibliometric analysis and systematic mapping were conducted using data from the Web of Science Core Collection and PubMed. Literature was retrieved using terms related to “stroke,” “brain-computer interface,” and “upper limb rehabilitation.” Screening followed the PRISMA guidelines. Visualization and quantitative mapping were performed using CiteSpace (v.6.4.R2) and VOSviewer (v.1.6.20) to evaluate publication volume, international collaboration, and keyword co-occurrence clusters.

**Results:**

Annual publications increased steadily from 37 in 2016 to 104 in 2025, with 65.6% published since 2020. The United States (*n* = 144), China (*n* = 83), and Italy were the most productive countries. Keyword analysis revealed a paradigm shift from functional electrical stimulation toward robotics-assisted therapy, motor imagery, and AI-driven decoding. Significant burst strengths were observed for “closed-loop systems,” “generative AI,” and “multi-modal feedback,” indicating these as the current primary frontiers.

**Discussion:**

BCI research for post-stroke recovery is transitioning from experimental signal processing to intelligent, multi-modal, and personalized clinical systems. Bibliometric evidence confirms that integrating BCI with robotic-assisted rehabilitation or functional electrical stimulation (FES) has become the mainstream clinical trend. Future efforts must focus on improving EEG signal stability and developing user-friendly hardware to facilitate the transition of BCI from research settings to daily clinical practice. China has emerged as the second most productive country, though international cooperation with European institutions remains an area for further growth.

## 1 Introduction

Stroke is a leading cause of long-term disability, with approximately 80% of patients experiencing persistent upper limb functional impairment. Despite intensive therapy, only 30% achieve significant functional recovery, imposing a severe socioeconomic burden on healthcare systems and families (GBD 2019 Stroke Collaborators, 2021). Consequently, developing innovative neurorehabilitation interventions remains a clinical priority.

Brain-computer interface (BCI) technology facilitates direct interaction between neural activity and external devices, bypassing damaged motor pathways (Wu et al., 2025). A standard non-invasive system typically integrates signal acquisition, feature extraction, and external control (Luo et al., 2022; Liu et al., 2025). In stroke rehabilitation, BCI utilizes motor imagery to activate cortical networks, creating a “central drive” that, when coupled with peripheral feedback such as functional electrical stimulation (FES) or robotics, establishes a closed-loop stimulation model to promote neuroplasticity (Biasiucci et al., 2018).

Over the past decade, research in this field has expanded rapidly, shifting from feasibility studies toward multimodal interventions and clinical translation. However, the proliferation of data makes it challenging for clinicians to identify key therapeutic trends. Bibliometric analysis offers a quantitative framework to visualize these evolutionary patterns (Chen et al., 2015). Existing reviews are often limited by narrow temporal ranges or single-database searches, failing to capture recent shifts like artificial intelligence integration.

The aim of this study was to systematically analyze English-language literature published between 2016 and 2025 regarding BCI for post-stroke upper limb rehabilitation. Utilizing CiteSpace and VOSviewer, we sought to delineate publication trends, research hotspots, and frontier trajectories to provide a strategic reference for future clinical practice and academic inquiry.

## 2 Materials and methods

### 2.1 Data sources and search strategy

This study was conducted in accordance with the Preferred Reporting Items for Systematic Reviews and Meta-Analyses (PRISMA) guidelines. A bibliometric and visualization analysis approach was employed to quantitatively assess the evolution of research on brain-computer interface (BCI) for post-stroke upper limb rehabilitation.

The literature retrieval was performed on March 9, 2026, across two primary databases: the Web of Science Core Collection (WoS) and PubMed. These databases were selected due to their comprehensive coverage of high-quality citation data and authoritative biomedical research, respectively. The search strategy was developed using a combination of Medical Subject Headings (MeSH) terms and free-text keywords. The detailed search strings for Web of Science Core Collection (WoSCC) and PubMed are presented in Table 1.

**TABLE 1:** Search strategy and strings.

Database	Search strategy
WoS	TS = ((“brain-computer interface” OR “brain-machine interface” OR BCI) AND (“upper limb*” OR “upper extremit*” OR arm OR hand OR “motor function”) AND (stroke OR “cerebrovascular accident*” OR poststroke OR hemiplegia) AND (rehabilitation OR recovery OR neurorehabilitation OR therapy))
PubMed	(((“brain-computervinterface” [Title/Abstract] OR “brain-computer interfaces”[Title/Abstract] OR “brain-machine interface”[Title/Abstract] OR BCI[Title/Abstract])) AND (“upper extremity”[Title/Abstract] OR “upper limb”[Title/Abstract] OR “arm”[Title/Abstract] OR “hand”[Title/Abstract] OR “motor function”[Title/Abstract]) AND (“stroke”[Title/Abstract] OR “cerebrovascular accident” [Title/Abstract] OR “hemiplegia”[Title/Abstract] OR “post-stroke”[Title/Abstract]) AND (“rehabilitation”[Title/Abstract] OR “recovery”[Title/Abstract] OR “motor recovery” [Title/Abstract]))

**FIGURE 1:**
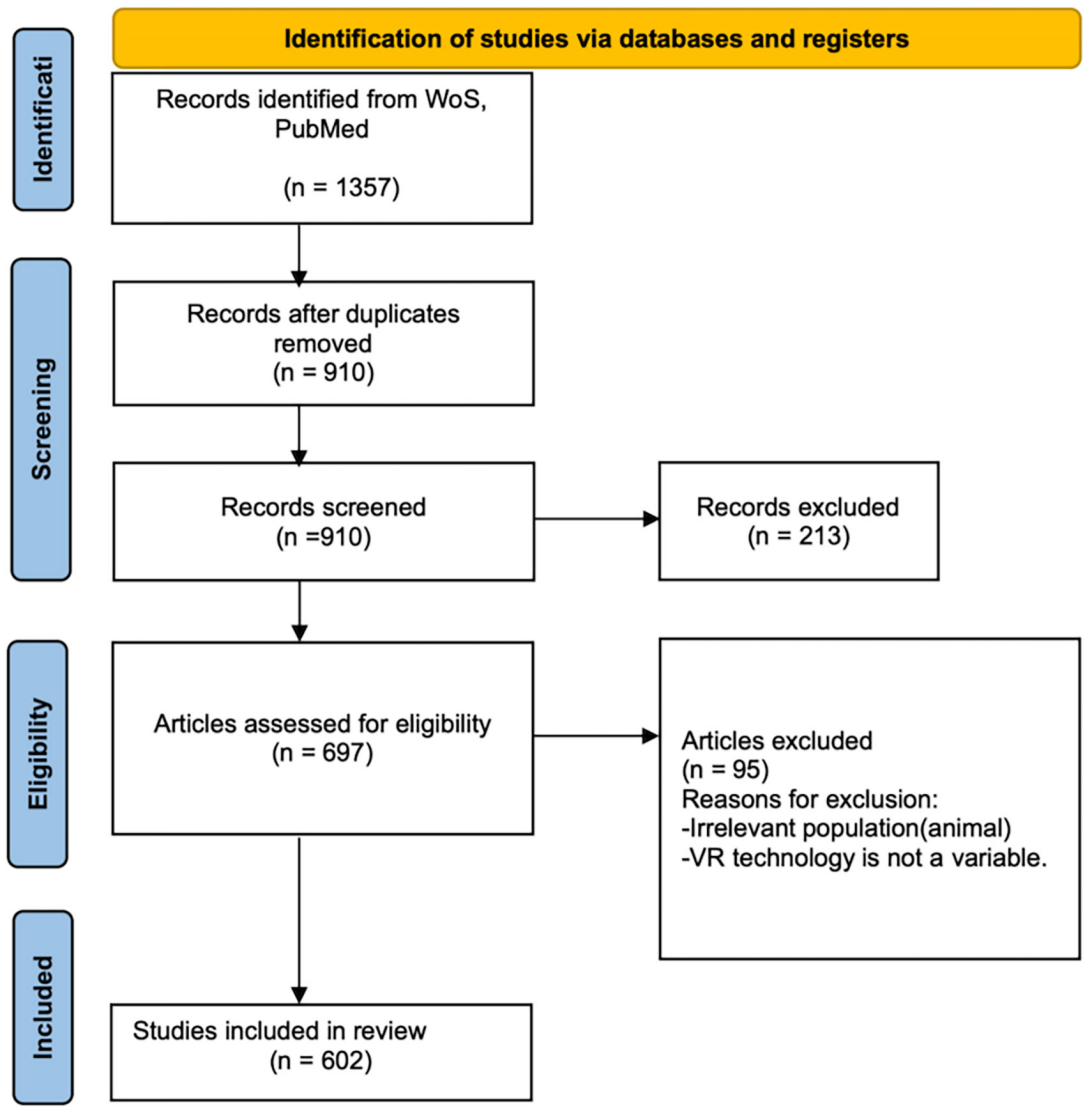
PRISMA flow diagram of the literature screening process.

The search period was restricted to publications between 2016 and 2025. Document types were limited to “articles” and “reviews” published in English. The initial search yielded 928 records from WoS and 429 from PubMed, totaling 1,357 records. Following the automated de-duplication workflow in CiteSpace and Zotero, exactly 447 duplicate records were identified and systematically removed, leaving 910 unique records. After a rigorous screening of titles, abstracts, and full texts based on the eligibility criteria, 602 publications were finalized for the final bibliometric analysis (Figure 1). Since the search was conducted in March 2026, the number of publications for that year remains limited and incomplete. Consequently, the study period was restricted to 2016–2025, excluding 2026 data to ensure the comparability and scientific validity of the annual publication trend analysis.

### 2.2 Inclusion and exclusion criteria

Studies were eligible for inclusion if they met the following criteria: (1) subjects were stroke survivors (ischemic or hemorrhagic); (2) BCI was utilized as a primary rehabilitation intervention; (3) outcomes measured upper limb functional improvement; (4) study designs were original research or reviews; and (5) the language was English.

Exclusion criteria included: (1) non-journal publications (e.g., meeting abstracts, dissertations, and patents); (2) studies where BCI was used solely for assessment rather than intervention; (3) animal experiments or basic laboratory research; and (4) studies where full-text access was unavailable.

### 2.3 Data analysis and visualization

Bibliometric analysis was executed using VOSviewer (version 1.6.20) (Eck and Waltman, 2010) and CiteSpace (version 6.3.1) (Xiao et al., 2011; Chen, 2008) within a Java environment. Prior to analysis, data were standardized to merge synonymous terms (e.g., “EEG” and “electroencephalography”) and reconcile redundant institutional names to ensure data integrity.

In CiteSpace, node types were sequentially analyzed (author, institution, country, and keyword) with a 1-year time slice. The g-index was applied as the selection criterion (*k* = 25), and pruning was performed via Pathfinder and Pruning Sliced Networks to enhance network clarity. In VOSviewer, minimum thresholds were set for authors (*n* = 2) and keyword occurrences (*n* = 2) to construct co-occurrence networks. Supplemental descriptive statistics were generated using Excel 2021.

## 3 Results

### 3.1 Analysis of publication volume

**FIGURE 2:**
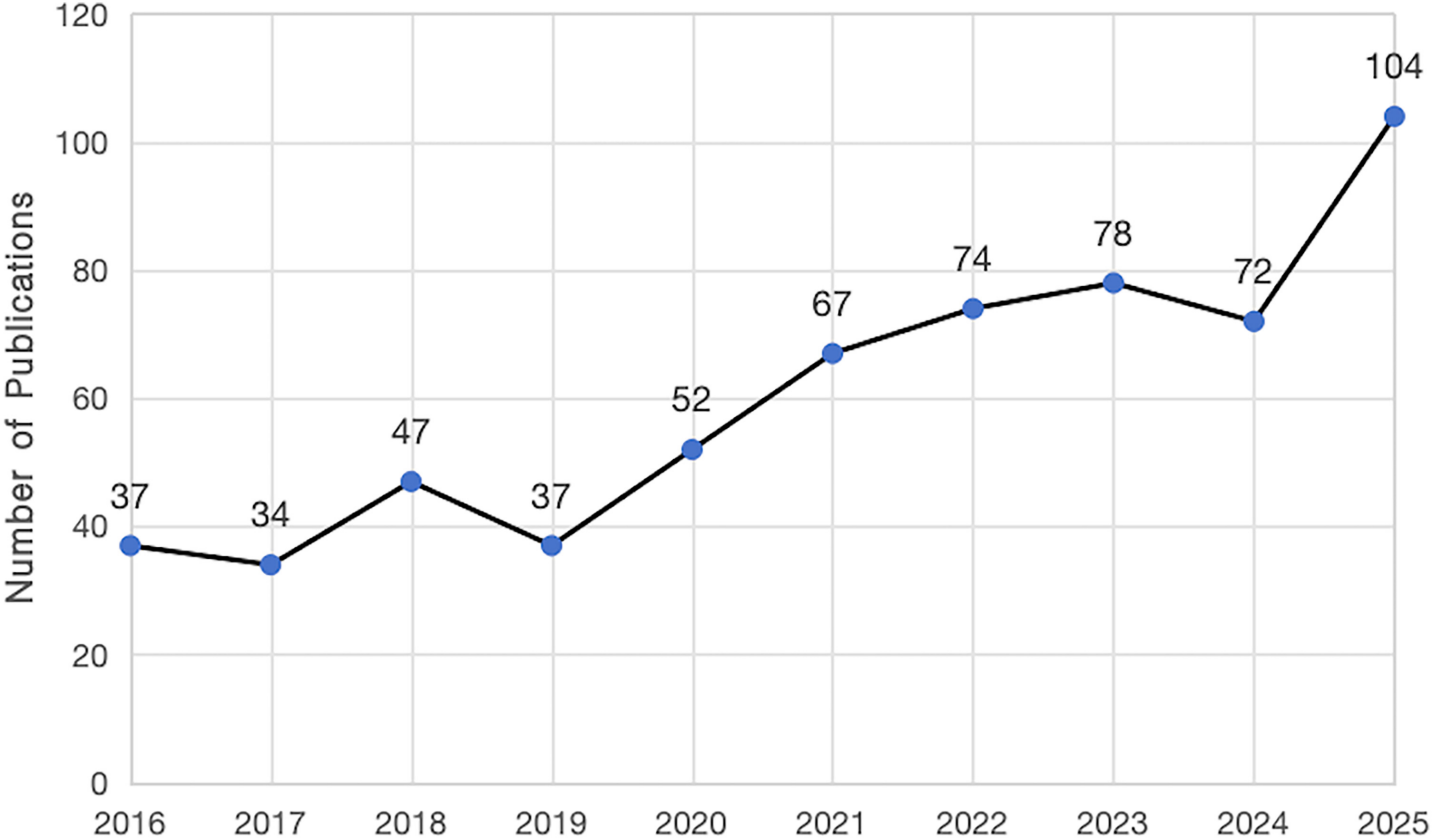
Annual publication status of literature. The bar chart represents the number of annual publications, while the line graph indicates the cumulative growth. Data were retrieved from the Web of Science Core Collection and PubMed.

The quantity and time span of literature publications reflect the academic development dynamics and evolutionary characteristics of a specific field (Yan and Zhiping, 2023). From 2016 to 2025, the annual number of publications in the field of BCI for post-stroke upper limb dysfunction showed an overall continuous growth trend (Figure 2). Regarding the growth trend, the volume in 2020 increased by 40.5% compared to 2016, and in 2023, it increased by 50.0% compared to 2020, indicating that the field has entered a period of rapid development over the past 5 years with a significant increase in research attention. In terms of cumulative publication volume, the number of publications in the last 5 years (2021–2025) was 395, accounting for 65.6% of the total volume over the past decade (602 articles), demonstrating the high volume and sustainability of research in this field. This upward trend may be related to the maturation of BCI technology, the popularization of portable EEG devices, and the continuous increase in demand for stroke rehabilitation. Further classification of the included literature indicates that the vast majority of studies predominantly focused on non-invasive BCIs, with Electroencephalography (EEG) serving as the primary technical route. This distribution closely aligns with clinical practice in rehabilitation medicine, where non-invasive BCI configurations are preferred for post-stroke upper limb recovery due to their superior safety profile, lower cost, and high feasibility in routine clinical settings.

### 3.2 Analysis of cited journals

As shown in Table 2, among the top 10 journals by citation frequency, Movement Disorders ranks first with 255 citations. This journal focuses on movement disorder diseases, reflecting the interdisciplinary nature of BCI in the field of motor rehabilitation. Parkinsonism and Related Disorders ranks second with 240 citations; it focuses on research related to Parkinson’s disease and associated movement disorders, indicating the versatility of BCI technology in the rehabilitation of neurological diseases. Journal of NeuroEngineering and Rehabilitation (JNER) ranks third with 222 citations; it is a specialized journal in the field of neuroengineering and rehabilitation and serves as a major hub for BCI rehabilitation research.

**TABLE 2:** Top 10 cited journals by citation frequency.

Rank	Cited journal	Citations
1	Movement Disorder	255
2	Parkinsonism Relat D	240
3	J Neuroeng Rehabil	222
4	Arch Phys Med Rehab	215
5	Plos One	214
6	PHYS THER	201
7	Neurorehab Neural Re	195
8	Gait Posture	186
9	Neurology	185
10	Clin Rehabil	178

**FIGURE 3:**
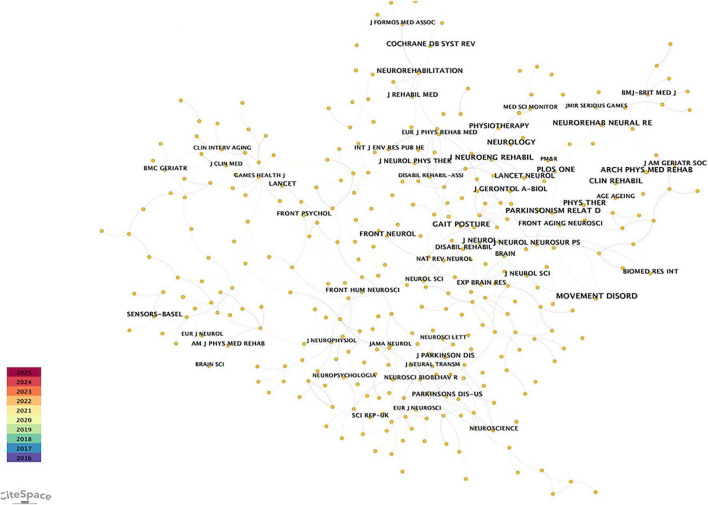
Network of cited journals. Each node represents a journal. The node size is proportional to the total number of citations the journal received within the analyzed dataset. The links between nodes represent co-citation relationships (i.e., two journals cited together in the same article). Purple rings indicate journals with high betweenness centrality, acting as intellectual bridges between different disciplines (e.g., merging neuroscience and clinical rehabilitation).

The cited journal network map (Figure 3) reveals a tight co-citation relationship between rehabilitation journals (e.g., Archives of Physical Medicine and Rehabilitation, Clinical Rehabilitation) and neuroengineering journals (e.g., Journal of NeuroEngineering and Rehabilitation, IEEE Transactions on Neural Systems and Rehabilitation Engineering), demonstrating the multi-disciplinary integration characteristics of this field. Furthermore, top-tier neurology journals (e.g., Neurology, Stroke) occupy central positions in the co-citation network, indicating the foundational neuroscience support for BCI rehabilitation research.

### 3.3 Analysis of country cooperation networks of authors

Among the top 10 countries by publication volume, the United States ranks first with 144 articles, followed by China with 83 articles and Italy with 79 articles, indicating that the United States has conducted more in-depth research in this field (Table 3).

**TABLE 3:** Top 10 countries by publication volume.

Rank	Country	Publications
1	USA	144
2	China	83
3	Italy	79
4	Germany	52
5	Australia	37
6	Spain	37
7	Brazil	34
8	Canada	34
9	Israel	24
10	Netherlands	20

**FIGURE 4:**
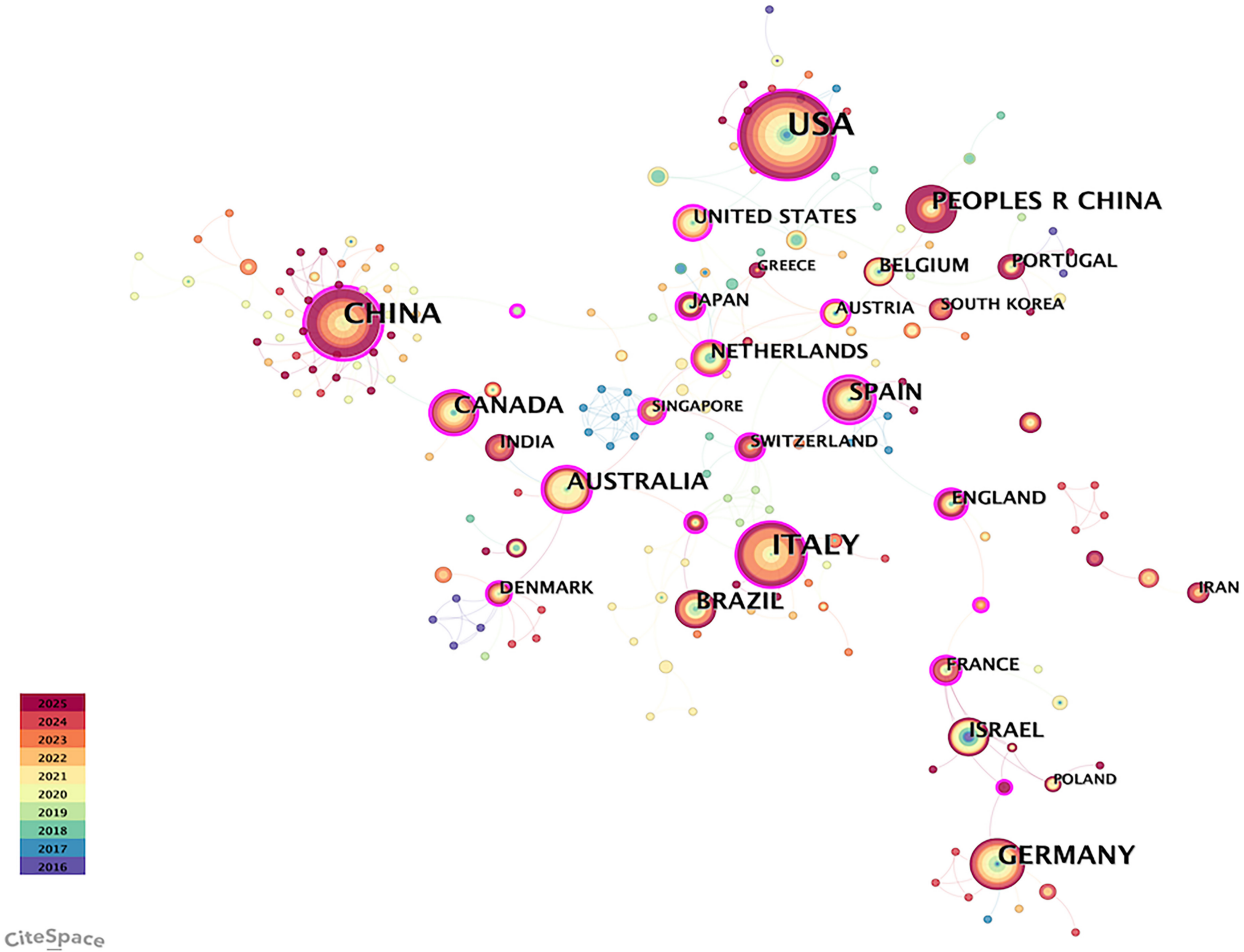
Map of country cooperation network. Each node represents a country or region. The size of the node is proportional to the number of publications in the field of BCI-based stroke rehabilitation. The lines (edges) connecting the nodes represent collaborative research relationships, with the thickness of the lines indicating the frequency of cooperation. Nodes surrounded by purple rings exhibit high betweenness centrality ( ≥ 0.1), identifying them as key hubs in the global BCI research network.

The country cooperation network map (Figure 4) shows that the United States has formed core cooperation clusters with countries such as China, Germany, Italy, Canada, and Australia. As the central node of the cooperation network, the United States maintains collaborative relationships with multiple countries, reflecting its leading position in global BCI rehabilitation research. Strong cooperation networks are evident among European countries (e.g., Germany, Italy, Spain, the Netherlands, and Switzerland). While China has stable cooperative relationships with countries such as the United States, Australia, and Singapore, its cooperation intensity with European countries is relatively low, suggesting room for expansion. This distribution characteristic indicates that research cooperation networks in North America and Europe are relatively mature. Although Asian countries have a high volume of publications, their international cooperation networks still need further improvement.

### 3.4 Analysis of authors

Among the top 10 authors by publication volume (Table 4), the German scholar Birbaumer Niels ranks first with 16 articles. His research focuses on BCI systems and neurofeedback for severely paralyzed patients, with a particular emphasis on BCI applications for patients with locked-in syndrome. The Chinese scholar Jia Jie ranks second with 14 articles, specializing in BCI combined with rehabilitation robotics and clinical rehabilitation applications (Jia, 2025). Singaporean scholars Guan Cuntai and Ang Kai Keng each published 14 articles, tied for second place, primarily focusing on BCI algorithms, motor imagery signal processing, machine learning classifier optimization, and clinical validation (Ang et al., 2011).

**TABLE 4:** Top 10 authors by publication volume.

Rank	Author	Country	Publications
1	Birbaumer, Niels	Germany	16
2	Jia, Jie	China	14
3	Guan, Cuntai	Singapore	14
4	Ang, Kai Keng	Singapore	14
5	Gharabaghi, Alireza	Germany	12
6	Ramos-murguialday, Ander	Spain/Germany	12
7	Cantillo-negrete, Jessica	Mexico	12
8	Ming, Dong	China	11
9	Soekadar,Surjo R.	Germany	11
10	Chen,Shugeng	China	11

**FIGURE 5:**
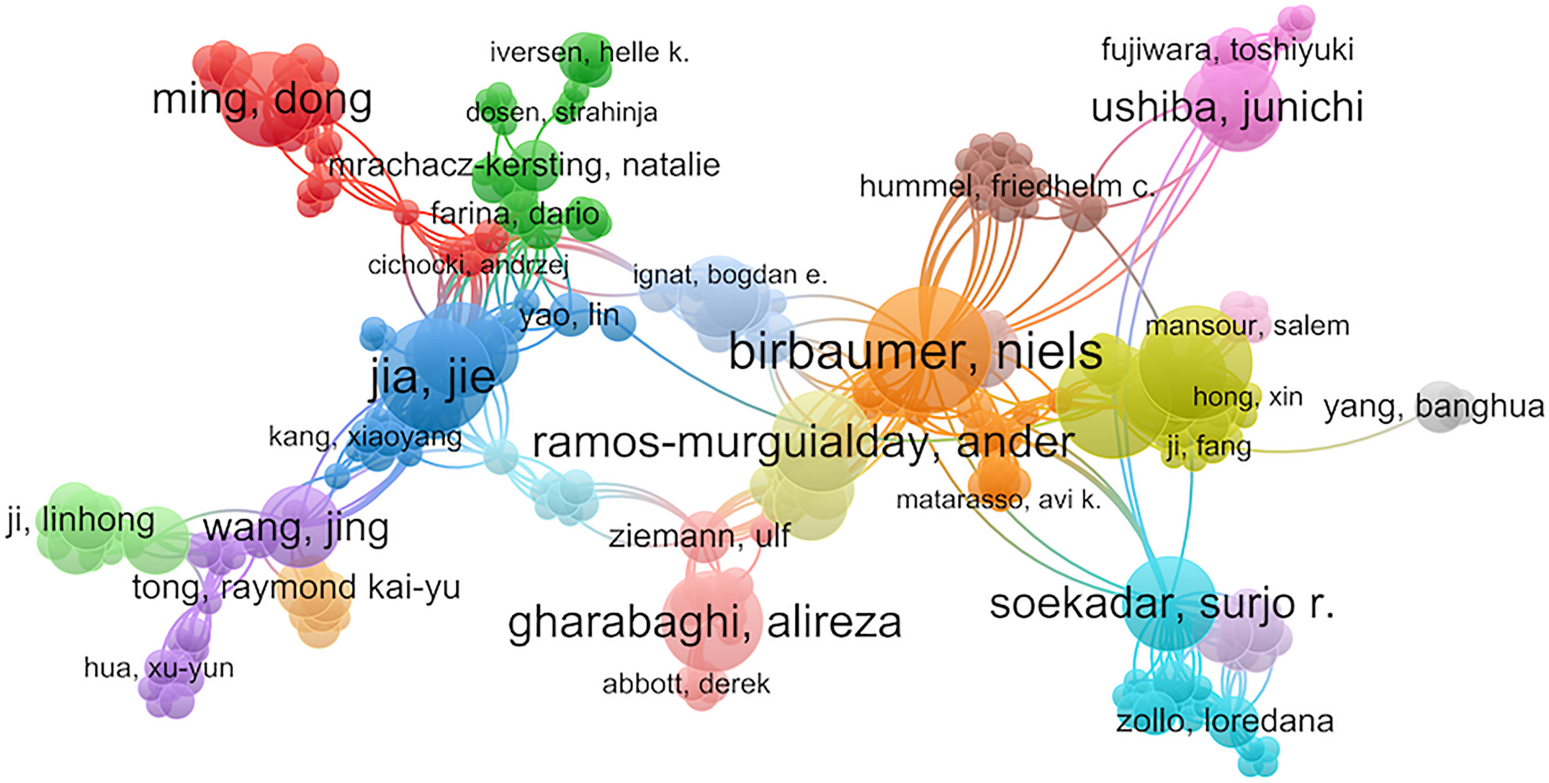
Author cooperation network. Each node represents an author. The size of the node indicates the author’s publication volume. The lines connecting the nodes represent co-authorship, with the distance between nodes reflecting the strength of their collaboration (shorter distances indicate stronger ties). Different colors represent clusters of authors who frequently collaborate, indicating distinct research groups or “invisible colleges” in the field.

The author cooperation network map (Figure 5) reveals four major research clusters: (1) A Singaporean team centered on Guan Cuntai and Ang Kai Keng, collaborating closely with institutions such as the National University of Singapore and Nanyang Technological University; (2) A European team centered on Birbaumer Niels and Ramos-murguialday Ander, with members distributed across Germany, Spain, Italy, and other countries; (3) A Chinese team centered on Jia Jie, Ming Dong, Chen Shugeng, and others, mainly concentrated in institutions such as Fudan University, Shanghai Jiao Tong University, and Tianjin University; (4) A German team centered on Gharabaghi Alireza, specializing in neuromodulation research involving BCI combined with TMS. Inter-regional cooperation exists between some teams; for instance, the Singaporean and German teams have joint publications in BCI algorithms and clinical trials, reflecting the research trend of international collaboration.

### 3.5 Analysis of research institutions

As presented in Table 5, the institutional hierarchy reveals that Tel Aviv University occupies a distinct and unparalleled leading position worldwide, with an aggregated total of 50 publications. Other globally prominent institutions follow in structural distribution, including the University of Sydney (16 publications) and the Universidade de Sao Paulo (14 publications). These are followed by active research hubs such as Rush University (9 publications), the University of Waterloo (8 publications), and Radboud University Nijmegen (8 publications), with the University System of Ohio (7 publications), IRCCS Bonino Pulejo (7 publications), Case Western Reserve University (6 publications), and Sapienza University Rome (6 publications) completing the elite global top 10.

**TABLE 5:** Top 10 institutions by publication volume.

Rank	Institution	Publications
1	Tel Aviv University	50
2	University of Sydney	16
3	Universidade de Sao Paulo	14
4	Rush University	9
5	University of Waterioo	8
6	Radboud University Nijmegen	8
7	University System of Ohio	7
8	IRCCS Bonino Pulejo	7
9	Case Western Reserve University	6
10	Sapienza University Rome	6

**FIGURE 6:**
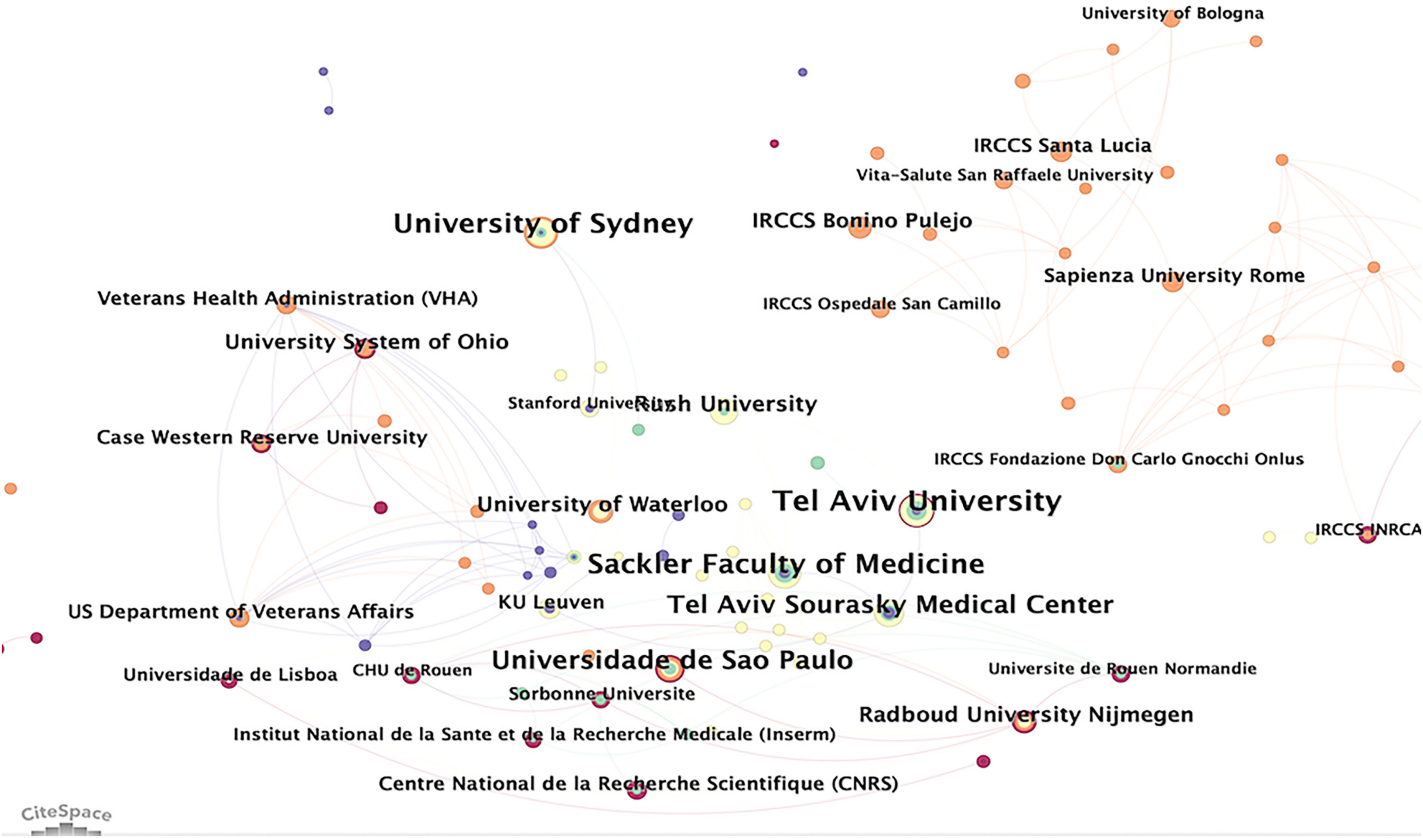
Cooperation network of research institutions. Each node represents a research institution (university, hospital, or research center). The size of the node is proportional to the institution’s publication volume. The lines (edges) between nodes represent collaborative relationships. Purple rings indicate institutions with high betweenness centrality ( ≥ 0.1), suggesting their role as key academic hubs that connect multiple research clusters.

The institutional cooperation network map (Figure 6) further reveals two distinct collaborative paradigms driven by these top organizations. First, Tel Aviv University represents a highly centralized, translational cluster, establishing a tight symbiotic alliance with its primary academic arm and major teaching affiliate, the Tel Aviv Sourasky Medical Center. This localized framework forms a complete pipeline that efficiently translates basic BCI algorithms into clinical stroke protocols. Second, the University of Sydney exemplifies a decentralized, multi-center clinical network, serving as a central hub that collaborates widely with multiple regional rehabilitation centers. This topology reflects an evidence-based research model driven strictly by diverse clinical problem-solving, maximizing patient cohort heterogeneity to validate BCI therapeutic outcomes.

### 3.6 Analysis of keywords

High-frequency keywords reflect research hotspots, and a centrality ≥ 0.1 is considered to indicate high influence (Wang et al., 2025). From a methodological perspective, betweenness centrality is a fundamental graph-theory metric in bibliometric studies that quantifies the degree to which a specific node acts as a strategic “informational bridge” or an intermediary highway between other disjointed clusters in the network. Within a clinical context, keywords or institutions with high betweenness centrality ( ≥ 0.1) do not merely signify high publication volume; instead, they represent critical multidisciplinary intersections where distinct medical or technical domains converge.

As shown in Table 6, structural mapping demonstrates a highly cohesive research core. “Brain computer interfaces” ranks first in frequency (*n* = 40, Centrality = 1.07), acting as the absolute foundation of the network, followed closely by “electroencephalography” (*n* = 23, Centrality = 0.68), “stroke” (*n* = 22 Centrality = 0.32), and “stroke rehabilitation” (*n* = 16, Centrality = 0.49). Crucially, keywords representing key technological and operational substrates—such as “imagery,” “algorithms,” “signal processing,” and “robotics”—exhibit exceptionally high betweenness centrality despite lower absolute frequencies. This topological distribution indicates that these technical metrics function as vital multidisciplinary highways, directly bridging engineering principles with clinical recovery workflows like “rehabilitation” and physical “movement.”

**TABLE 6:** Top 11 keywords of the literature.

Rank	Keyword	Centrality	Frequency
1	Brain computer interfaces	1.07	40
2	Electroencephalography	0.68	23
3	Stroke	0.32	22
4	Stroke rehabilitation	0.49	16
5	Imagery	0.41	11
6	Rehabilitation	0.10	8
7	Signal processing	0.23	6
8	Movement	0.14	6
9	Robotics	0.20	6
10	Algorithms	0.44	5

#### 3.6.1 Keyword co-occurrence analysis

**FIGURE 7:**
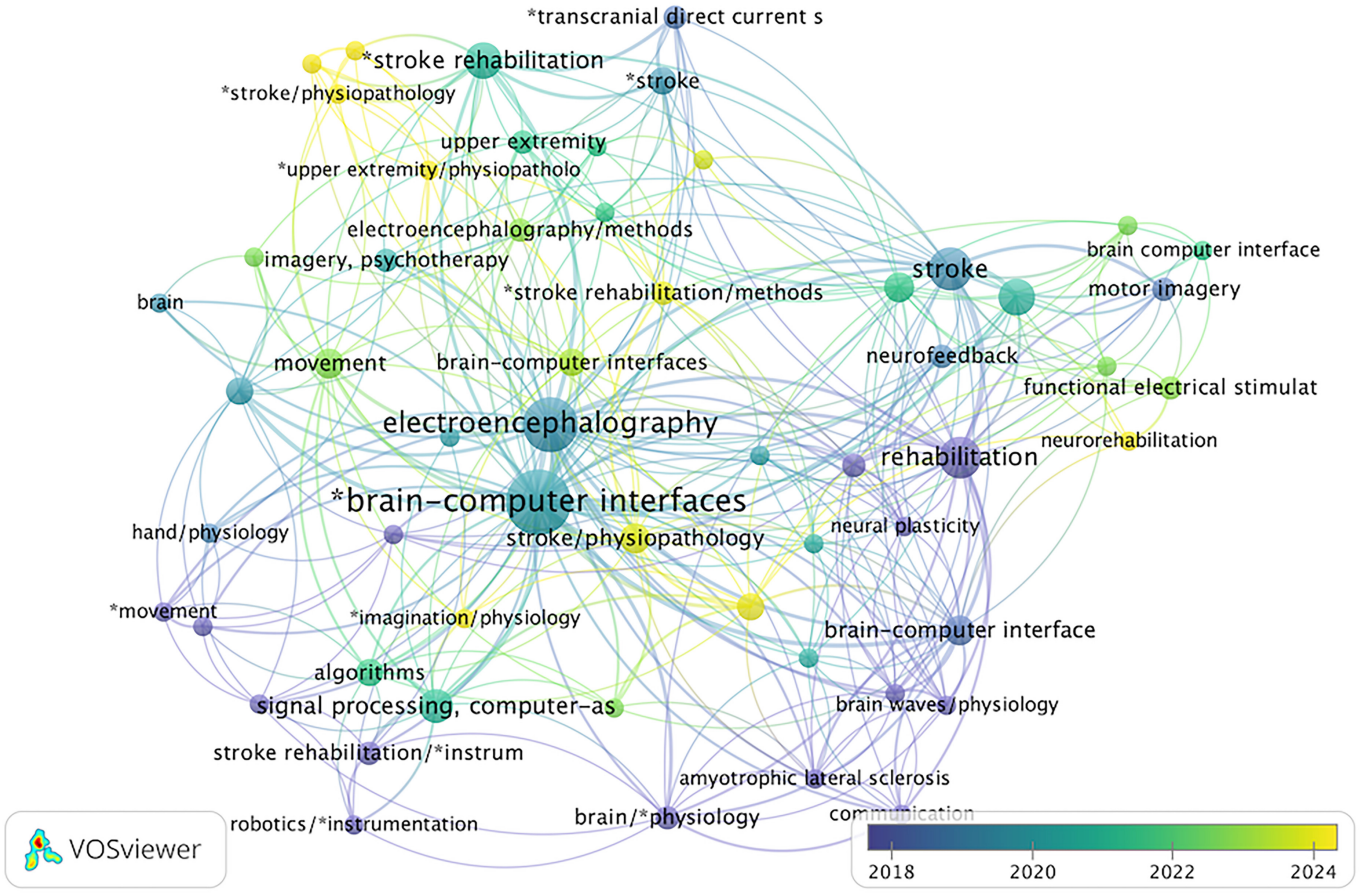
Visualization map of keyword co-occurrence. Each node represents a keyword. The size of the node and the font size of the label are proportional to the frequency of the keyword’s occurrence. The distance between two nodes reflects the strength of their relationship; a shorter distance indicates a higher co-occurrence frequency. Different colors categorize keywords into distinct clusters, representing major research themes and hotspots within the field.

VOSviewer software was utilized to analyze keywords in literature related to the improvement of upper limb dysfunction after stroke using brain-computer interfaces. Keywords with a frequency ≥ 5 were selected to explore research hotspots and trends in this field (Zhang et al., 2025). The co-occurrence network visualization map (Figure 7) shows that “brain computer interfaces” and “electroencephalograph” and “stroke” serve as the absolute core hubs of the network. These central nodes extend outward to interconnect heavily with operational keywords, including “motor imagery” “stroke rehabilitation” “functional electrical stimulation” “robotics” “neurofeedback” and “signal processing” thereby forming multiple research networks. Motor imagery (as a BCI control paradigm), electroencephalographic signal processing (as technical support), and functional electrical stimulation combined with rehabilitation robotics (serving as the primary physical feedback mechanisms) constitute the three structural pillars of contemporary research. Furthermore, peripheral therapeutic nodes such as “robotics,” “neurofeedback,” and “transcranial direct current stimulation (tDCS)” remain highly integrated with the central clusters. The overlay chronological coloring (from 2018 to 2024) visually documents a progressive and ongoing evolutionary trend, transitioning from basic electrophysiological signal validation toward highly synergistic, multi-modal combined interventional strategies directly tailored for real-world stroke rehabilitation and upper extremity functional tracking.

#### 3.6.2 Keyword clustering analysis

Keyword clustering analysis was conducted using the log-likelihood ratio (LLR) algorithm in CiteSpace (Yang, 2021).

**FIGURE 8:**
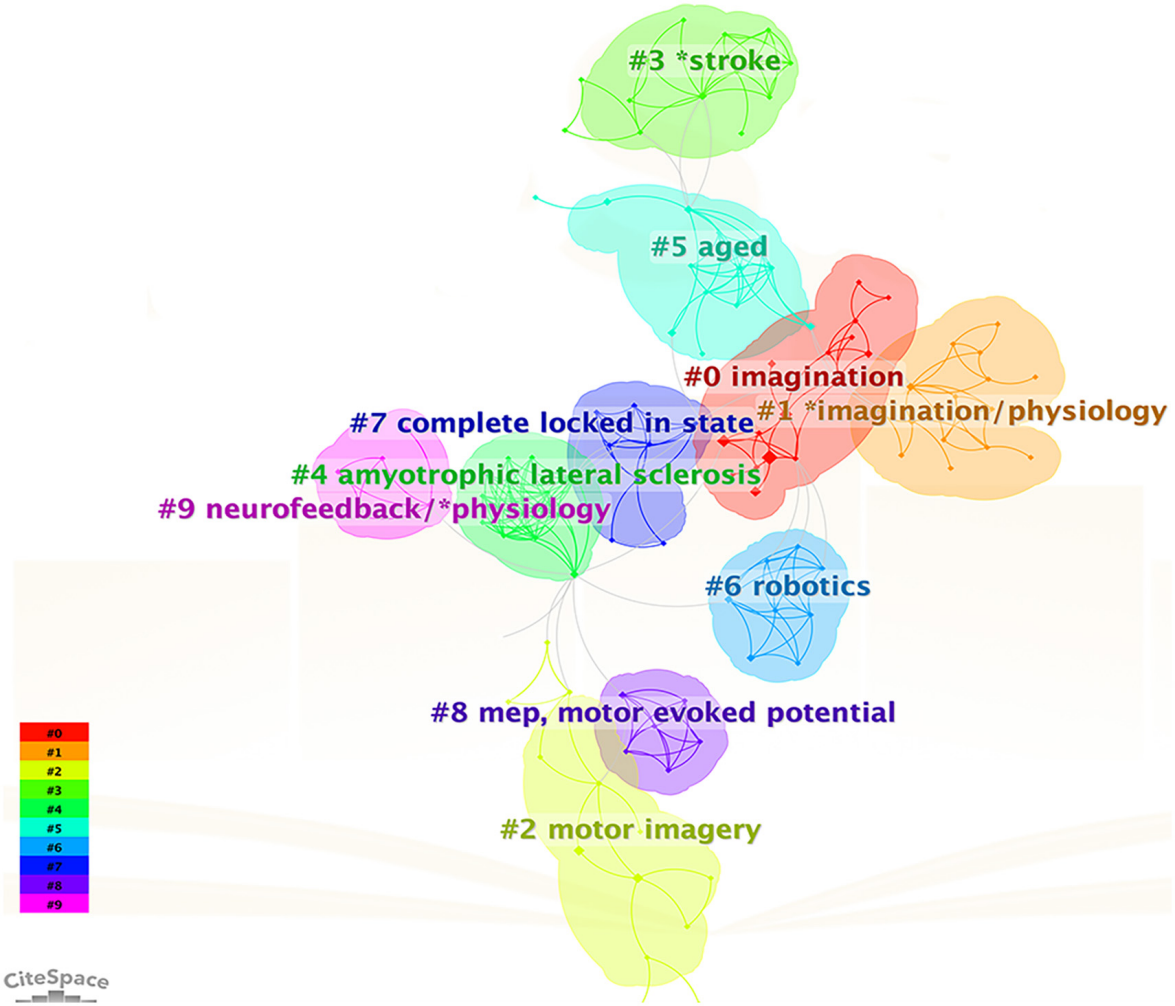
Visualization map of keyword clustering analysis.

A total of 10 clusters (#0 to #9) were formed. The keyword clustering map (Figure 8) shows a modularity value (Q) of 0.7998 and a mean silhouette value (S) of 0.9695. Generally, the Q value ranges within [0, 1); a Q > 0.3 indicates a significant community structure, and an S value above 0.5 is considered to represent a reasonable clustering (Ding et al., 2025). Based on the relevance of the research topics, the 10 clusters can be categorized into four major research themes: core motor imagery BCI paradigms and signal processing techniques (#0, #1, #2, #5, #8), multi-modal combined intervention strategies (#6, partial #8), clinical applications and functional translation (#3, partial #6), and applications for specific populations and user experience design (#4, #7, #9). Table 7 presents the number of nodes, silhouette values (S), and primary keywords (LLR) for each cluster.

**TABLE 7:** Information of 10 representative keyword clusters.

Cluster ID	Size	Silhouette (S)	Major keywords (LLR)
#0	16	1	imagination; hand/physiology; brain; imagery, psychotherapy; *stroke/diagnosis
#1	15	0.985	*imagination/physiology; signal processing, computer assisted; algorithms; *learning; spectroscopy, near infrared/*instrumentation
#2	13	0.964	motor imagery; brain computer interface; functional electrical stimulation; stroke; motor function recovery
#3	13	1	*stroke; hand strength; intention; motor activity; activities of daily living
#4	13	0.96	amyotrophic lateral sclerosis; ecog = electrocorticography; tetraplegia; bci; bci = brain–computer interface
#5	13	0.907	aged; middle aged; *imagination; *stroke/physiopathology; *imagery, psychotherapy/methods
#6	8	0.968	robotics; neurofeedback; hemiplegia; imagery, psychotherapy/*methods; treatment outcome
#7	8	0.94	complete locked in state; conditioning, classical/physiology; chronic disease; *communication; functional near infrared spectroscopy
#8	6	1	mep, motor evoked potential; transcranial magnetic stimulation; severe hemiplegia; tms, transcranial magnetic stimulation; bmi
#9	5	0.979	neurofeedback/*physiology; user centered design; communicable diseases/*etiology/*rehabilitation; eeg; brain injuries/*complications/rehabilitation

#### 3.6.3 Keyword burst analysis

**FIGURE 9:**
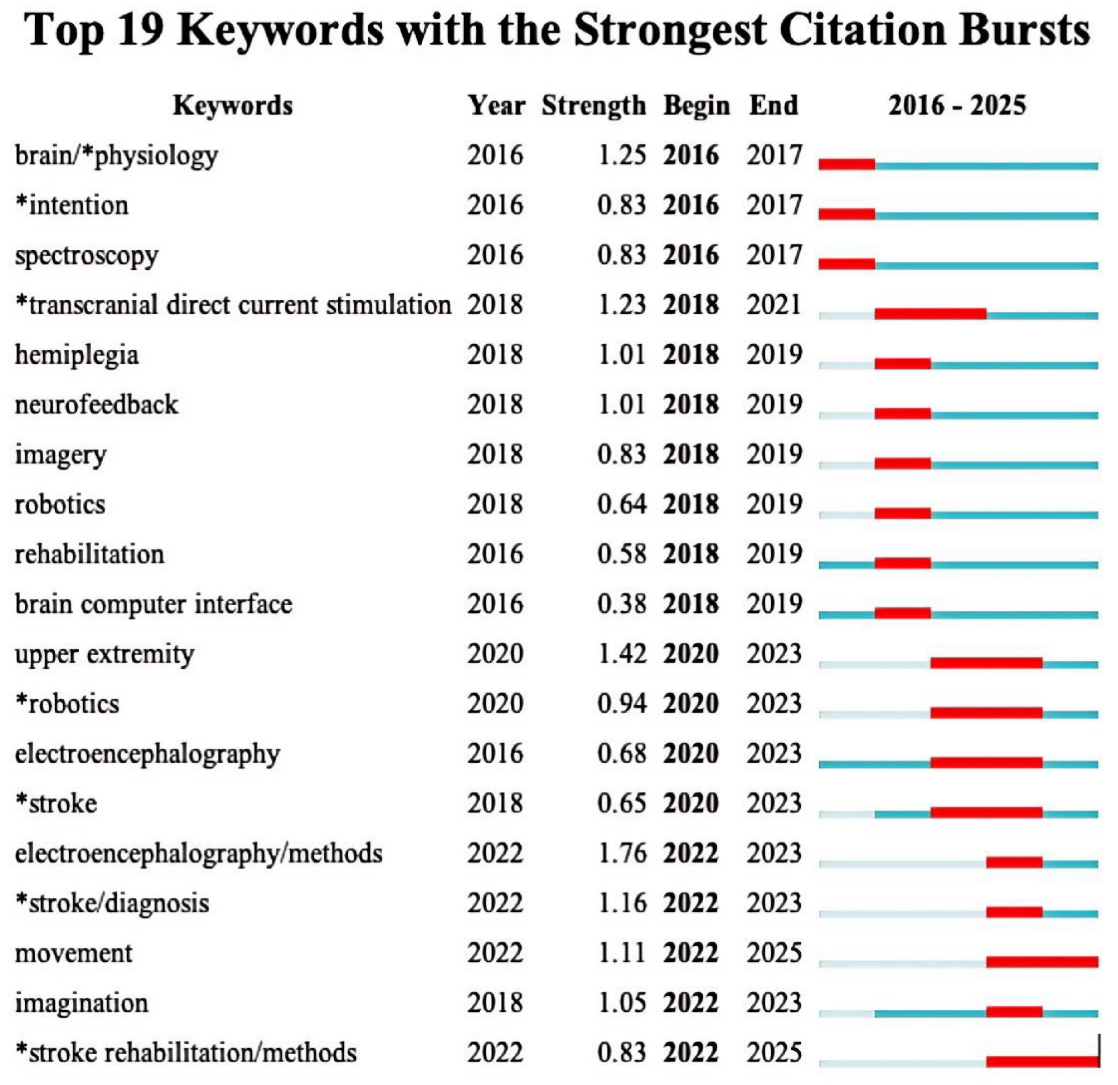
Visualization map of keyword burst detection. The blue lines represent the entire time span (2016–2025), and the red segments indicate the duration of the citation burst. “Strength” represents the intensity of the burst. Keywords ending with a red segment in 2025 represent the current research frontiers and future trends in the field of BCI stroke rehabilitation.

CiteSpace was used to analyze the keyword burst maps in both Chinese and English. A keyword burst reflects a sharp increase in research frequency within a short period, as shown in Figure 9.

To systematically trace the historical evolution and shifts in research hotspots, the keyword burst timeline was structured into three distinct chronological phases. Crucially, this temporal staging was established via a hybrid quantitative-qualitative workflow to eliminate subjective narrative bias. The precise chronological boundaries were mathematically demarcated based on the statistical inflection points of annual global publication growth rates and the chronological onset density of high-strength keyword bursts derived from the algorithms. Once these empirical data boundaries were structurally locked, the three descriptive thematic nomenclatures were applied to characterize the primary technological paradigms of each respective era.

The first stage (2016–2018): Early Exploration Period. The burst keywords during this stage included “brain/physiology” “intention” and “spectroscopy” Research in this period primarily focused on the neurophysiological basis of BCI, exploring the combined application of BCI with transcranial direct current stimulation (tDCS), neurofeedback, and robotics, with a focus on rehabilitation outcomes for hemiplegic patients. The second stage (2020–2023): Rapid Development Period. Burst keywords in this stage included “upper extremity” “robotics” “electroencephalography” and “stroke” During this period, “EEG methodology” became the keyword with the highest burst strength. Upper limb function received continuous attention as a core outcome measure, and the burst of robotic-assisted combined interventions persisted. Stroke diagnosis and rehabilitation methods became the research focus. The third stage (2022–2025): Continuous Frontier Period. Keywords with bursts continuing through 2025 include “movement” and “stroke rehabilitation/methods,” indicating that motor function rehabilitation methodology remains the current research frontier.

## 4 Discussion

The bibliometric data from 2016 to 2025 underscores an accelerating trajectory in BCI research for stroke rehabilitation, transitioning from early invasive paradigms focused on high-bandwidth neural decoding, such as the BrainGate project (Gusman et al., 2025), to sophisticated non-invasive clinical translations and integrated university-hospital academic frameworks (Lee et al., 2024). Our analysis highlights that global productivity is driven not only by strong national funding pipelines, but also by high-density localized research ecosystems. Notably, Tel Aviv University has emerged as a global leader in this domain (Table 5), utilizing an interconnected translational model to bridge basic algorithmic engineering with rapid clinical validation. Simultaneously, China’s rapid rise is inseparable from its strategic national focus, particularly through the “Intelligent Robots” special project under the National Key R&D Program and the “Brain Science and Brain-Like Research” major project (Tianjin University, 2026; National Natural Science Foundation of China, 2025). While China ranks second in publication volume, the density of its international collaboration network remains relatively sparse compared to the tight-knit, high-density collaborative alliances observed in leading Mediterranean and Western clusters (Figure 6). Given the established clinical traditions and unified institutional ecosystems in these leading regions, strengthening cross-border and multi-center collaborative networks is essential for establishing evidence-based global protocols for BCI implementation. This macro-level integration suggests that the future landscape of stroke rehabilitation will increasingly rely on the structural convergence of refined non-invasive interfaces, robust machine learning workflows, and highly standardized clinical execution pathways.

A key theme emerging from the keyword co-occurrence analysis is the superiority of multimodal intervention strategies. Motor imagery (MI) remains the core control paradigm, leveraging the significant neuro-activation overlap between imagery and execution in cortical regions such as the primary motor cortex (M1), premotor area (PMA), and supplementary motor area (SMA) (Hétu et al., 2013). This neurophysiological foundation is critical for patients with severe upper limb paralysis who lack residual electromyographic (EMG) signals (Ang and Guan, 2015). When evaluating research influence beyond mere publication volume, citation quality indicators reveal that the academic trajectory of this field is anchored by several highly cited landmark studies that define its historical H-index boundaries. The integration of MI-BCI with functional electrical stimulation (FES) and rehabilitation robotics (Remsik et al., 2022; Wu, 2025; Ji et al., 2025) is conceptualized to facilitate an “intention–execution–feedback” closed-loop. This structural paradigm shift is structurally epitomized by the landmark clinical trial conducted by Biasiucci et al. (2018), a highly cited cornerstone showing that brain-actuated FES elicits lasting, clinically significant upper-limb motor recovery by driving targeted neuroplasticity—a study that remains a major driver of the field’s average citations per article. Within the literature framework, this structural integration is hypothesized to align with the “Hebbian plasticity” principle, suggesting that the central motor intention (top-down) precisely synchronized with peripheral somatosensory feedback (bottom-up) may contribute to the enhancements observed in Fugl-Meyer assessment scores and Activities of Daily Living (ADL) compared to single-mode therapies (Chen and Yun, 2026).

The clinical efficacy of long-term BCI training is powerfully demonstrated by the Walk Again Project (WAP). By combining non-invasive BCI with virtual reality and exoskeletons, the WAP protocol enabled 50% of chronic spinal cord injury patients to improve their neurological status from ASIA A to ASIA C (Donati et al., 2016; Shokur et al., 2018). Notably, significant functional recovery was observed even in patients 20 years post-injury, which has been interpreted within the field as preliminary evidence that BCI-driven closed-loop feedback might potentially trigger late-stage neuroplasticity. This line of high-influence research has culminated in recent premium-tier breakthroughs, such as the digital bridge engineered by Lorach et al. (2023) in Nature, which restored natural walking after spinal cord injury via a brain-spine interface. As a bibliometric evaluation, our analysis captures these widespread, highly cited landmark milestones as the dominant quality-defining vehicles guiding contemporary publication trends, demonstrating that global research influence is heavily concentrated within these heavily-vetted, high-impact clinical translation frameworks, though large-scale randomized controlled trials remain essential to fully validate these empirical neurophysiological pathways.

The research frontier is shifting toward intelligence and portability. The emergence of hybrid fNIRS-EEG acquisition systems (Medvedeva et al., 2026) marks a milestone; by utilizing the higher resistance to motion artifacts and superior spatial resolution of fNIRS (Meng et al., 2025), clinicians can obtain more stable metabolic and electrophysiological data in real-world settings. Within this landscape, the overwhelming statistical dominance of electroencephalography (EEG) identified in our bibliometric profile should be interpreted with methodological caution; this high publication volume reflects the modality’s superior clinical feasibility, cost-effectiveness, and non-invasive safety rather than absolute therapeutic or technological superiority over alternative or invasive interfaces. EEG-based systems remain highly affordable and practical for iterative multi-session stroke rehabilitation, bypassing the surgical risks and institutional costs associated with high-bandwidth neural implants, which naturally inflates its representation in current research output. To overcome the long-standing “small sample” bottleneck caused by individual cortical lesion differences in stroke patients, generative AI, including Diffusion models and GANs (Dere et al., 2026; Xu et al., 2022), is being used to synthesize high-quality EEG samples, thereby enhancing classifier robustness. Furthermore, the integration of neuromorphic computing and spiking neural network (SNN) chips (Yao et al., 2024) offers potential for ultra-low-power, real-time signal processing.

Looking forward, the prominent and ongoing citation bursts of “movement” (2022–2025, Strength = 1.11; Figure 9) and “stroke rehabilitation/methods” (2022–2025, Strength = 0.83; Figure 9) indicate a critical paradigm shift toward methodological optimization for practical physical recovery. Accompanied by the structural link between Cluster #2 (motor imagery) and Cluster #6 (robotics), the primary research frontier is transitioning from basic brain-signal identification to highly targeted clinical execution protocols. Future efforts should focus on refining closed-loop neuromodulation methods that map motor-imagery intent directly onto specific physical movement coordinates. By syncing advanced electroencephalography processing with real-time robotic adjustments, these methodologies aim to deliver highly precise, personalized physical feedback. This data-driven approach directly translates the quantitative trends identified in our bibliometric maps into concrete clinical strategies for targeted neuroplasticity induction. Furthermore, the presence of peripheral terms within our keyword clustering network, such as “amyotrophic lateral sclerosis” (ALS), “tetraplegia,” and “communicable diseases,” merits methodological contextualization, as they reflect genuine interdisciplinary spillover rather than data extraction noise (Ali et al., 2025). Historically, the fundamental algorithmic frameworks utilized in post-stroke upper-limb BCI rehabilitation, including sensory-motor rhythm tracking and advanced machine learning classifiers, were initially engineered and validated in patients suffering from total motor paralysis, including ALS and traumatic spinal cord tetraplegia (Nuyujukian et al., 2018). The persistent co-occurrence of these conditions underscores a shared technical lineage where stroke rehabilitation directly adapts foundational signal-decoding technologies from severe neurodegenerative models. Parallelly, the clustering of “communicable diseases” captures a crucial environmental pivot during the COVID-19 pandemic era (Bonanno et al., 2025). The severe restrictions placed on traditional, in-hospital physical therapy during this public health crisis served as a powerful catalyst for the field, accelerating the development and clinical adoption of decentralized BCI technologies (Mansour et al., 2025). This shift heavily prioritized home-based BCI systems, tele-rehabilitation frameworks, and contactless virtual-reality-guided motor training. Thus, these seemingly unrelated terms effectively document the technical ancestry and the socio-environmental factors that continue to shape the contemporary clinical boundaries of BCI-driven stroke recovery.

The findings of this bibliometric analysis offer several practical insights and prospective implications for rehabilitation clinicians. Firstly, the paradigm shift from traditional FES-BCI to multi-modal closed-loop systems suggests that clinicians might consider evaluating integrated platforms that combine motor imagery with synchronous sensory feedback (e.g., robotic gloves or haptic feedback) as prominent research paradigms optimized for fostering neuroplasticity. Secondly, the emergence of AI-driven adaptive BCI indicates a technological movement toward conceptual “precision rehabilitation.” Clinicians can anticipate the progressive development of potential tools that dynamically adjust training difficulty based on a patient’s real-time cognitive load and fatigue, thereby theoretically aiming to reduce “BCI-illiteracy” and improving therapy adherence. Lastly, the significant clinical recovery (ASIA A to C) compelling data from long-term clinical protocols provides a strong incentive to explore extending BCI interventions into the chronic phase of stroke, offering a fresh perspective that challenges the traditional “recovery plateau” concept. Crucially, as these observations are derived from literature distribution matrices rather than primary randomized controlled trials, they should be treated as directional trends within the academic community rather than definitive prescriptive guidelines for clinical efficacy.

Despite the comprehensive nature of this bibliometric study, certain limitations must be acknowledged. The reliance on English-language literature from WoS and PubMed may lead to the omission of significant clinical insights published in other languages. Furthermore, while our search syntax comprehensively targeted standard descriptors, certain peripheral terminologies—such as “neural interface,” or “assistive neurotechnology,” or “brain-machine rehabilitation”—were not explicitly itemized, potentially excluding a minority of niche reports. More importantly, the exclusion of databases such as Scopus, Embase, and IEEE Xplore introduces a potential selection bias. While merging distinct platforms was avoided to prevent metadata formatting inconsistencies and duplicate errors that compromise software clustering algorithms, the absence of IEEE Xplore may undersample engineering-oriented BCI conference proceedings, and the omission of Embase may limit slightly biomedical saturation. Additionally, it is critical to contextualize our inclusion of ultra-recent references spanning 2025–2026 naturally encompass early-stage preprints and technical reports. while capturing these datasets is necessary to capture the immediate contemporary frontier and sudden keyword bursts, within current database metrics, these source materials represent emerging technological concepts rather than established clinical evidence. readers should interpret these recent machine-learning trend spikes—such as generative sample synthesis or hybrid metabolic-electrophysiological modeling—with appropriate caution, as their long-term technical stability and clinical neurophysiological efficacy remain unverified by long-term peer review. Future bibliometric efforts will benefit from incorporating longitudinal citation windows that can systematically filter out temporary technical noise from genuinely sustainable clinical breakthroughs. Future bibliometric efforts will benefit from incorporating longitudinal citation windows, expanding database coverage to Chinese-language platforms (such as CNKI), and combining visualization maps with methodological quality assessments of randomized controlled trials (RCTs) to provide a clearer evidence-based roadmap for clinical adoption. Future research should prioritize the inclusion of Chinese-language databases (such as CNKI) and non-journal document types, including patents and doctoral dissertations, to capture a more complete picture of the intellectual property landscape and localized clinical trials. Moreover, while visualization tools map the structural trends of the field, they do not inherently reflect the methodological rigor of the primary studies. Future systematic reviews should combine bibliometric mapping with quality assessments of randomized controlled trials (RCTs) to provide a clearer evidence-based roadmap for clinical adoption.

## 5 Conclusion

BCI for post-stroke rehabilitation is evolving into an intelligent, multi-modal, and patient-centered discipline. The synergistic application of neuromodulation techniques like TMS (Luk et al., 2022; Meinel et al., 2021; Zhen et al., 2025), advanced AI architectures, and robotics is bridging the gap between experimental concepts and clinical practice. However, achieving widespread clinical integration requires overcoming hardware usability barriers and validating long-term functional outcomes through large-scale, transnational multi-center trials. Rather than serving as an immediate or standard component within the contemporary stroke rehabilitation toolkit, BCI technology currently remains in a critical translational phase. Its routine clinical implementation is heavily constrained by several unresolved systemic barriers, including high institutional deployment costs, complex and time-consuming signal calibration workflows, and high patient neurophysiological heterogeneity caused by diverse stroke lesion topologies. Addressing these technical, clinical, and financial friction points through large-scale evidence-based validation is an essential prerequisite before BCI can realistically transition from an experimental academic frontier into a reliable adjunctive modality in standard neurorehabilitation workflows, ultimately enhancing the quality of life for stroke survivors globally.

## Data Availability

The original contributions presented in the study are included in the article/supplementary material, further inquiries can be directed to the corresponding author.
